# Skin metastases of cervical cancer: two case reports and review of the literature

**DOI:** 10.1186/s13256-016-1042-0

**Published:** 2016-09-23

**Authors:** Meryem Benoulaid, Hanan Elkacemi, Imane Bourhafour, Jihane Khalil, Sanaa Elmajjaoui, Basma Khannoussi, Tayeb Kebdani, Noureddine Benjaafar

**Affiliations:** 1 Department of Radiotherapy, National Institute of Oncology, Mohammed V University, Rabat, Morocco; 2 Department of Pathology, National Institute of Oncology, Mohammed V University, Rabat, Morocco

**Keywords:** Case report, Carcinoma of the uterine cervix, Skin metastasis

## Abstract

**Background:**

Although cervix carcinoma is one of the most common malignancies in women, hematogenous metastases are relatively not common. Cutaneous metastases, in particular, are unusual even at an advanced stage of disease. Their presence is a predictor of poor prognosis.

**Case presentation:**

Case 1: A 63-year-old postmenopausal Moroccan woman was diagnosed as having cervical squamous cell carcinoma. She was treated with radical concurrent chemotherapy and radiation therapy followed by low-dose brachytherapy. Six months after finishing the therapy, multiple skin nodules appeared on her abdomen and chest wall. An excision biopsy was performed and showed metastatic squamous cell carcinoma. Her disease progressed and she died before completing her fourth course of palliative chemotherapy.

Case 2: A 48-year-old Moroccan woman was diagnosed as having cervical squamous cell carcinoma; she was treated with concurrent chemoradiation. Before a planned high-dose brachytherapy, she noticed many nodular lesions on her arms, thighs, and chest wall. An excision biopsy was performed and showed metastatic squamous cell carcinoma. She then underwent a series of imaging examinations, including computed tomography of her chest, abdomen, and pelvis, and a whole body bone scan that showed disseminated disease involving her lungs and bones. She died after two courses of palliative chemotherapy, 2 months after the appearance of the skin lesions.

**Conclusion:**

We report two cases to illustrate a rare localization of metastasis from cervical carcinoma that is highly aggressive requiring early detection and aggressive management.

## Background

Carcinoma of the uterine cervix is the most common gynecological cancer in developing countries. The most frequently reported metastases sites are lungs, bone, and liver [1, 2]. Skin metastases are extremely rare even in the late stages of disease; they have an incidence range from 0.1 to 2 % [3, 4]. The presence of metastatic disease in the skin is a strong predictive of poor prognosis, as skin metastases are usually associated with local or regional recurrence. The treatment is usually palliative with very poor outcomes. We report here the cases of two patients with locally advanced cervical cancer who developed cutaneous metastasis.

## Case presentation

### Case 1

Nine years ago a 63-year-old Moroccan woman was referred to our department with the complaint of postmenopausal bleeding and foul smelling vaginal discharge. A pelvic examination revealed a 4 cm cervical lesion extending to her upper vaginal wall with left parametrial extension. A biopsy indicated squamous cell carcinoma moderately differentiated and keratinizing of her cervix. Computed tomography of her chest, abdomen, and pelvis showed a cervical tumor of approximately 5 cm with hydroureteronephrosis. There was no evidence of distant metastasis. Consequently, she was staged as stage IIIB according to the International Federation of Gynecology and Obstetrics (FIGO) staging system. She received concurrent chemoradiation followed by low-dose brachytherapy.

Six months after finishing the treatment, she presented with multiple abdominal and thoracic subcutaneous nodules. A skin biopsy was performed and a diagnosis of metastatic squamous cell carcinoma was confirmed. She was then given palliative chemotherapy using a single drug protocol with carboplatin. However, the disease progressed, and she died before completing her fourth course of chemotherapy.

### Case 2

A 48-year-old Moroccan woman presented to our radiotherapy department, 2 years ago, with a 6-month history of vaginal bleeding. On examination, a 5 cm cervical lesion extending to the upper third of her vagina with bilateral parametrial involvement was identified. A cervical biopsy confirmed squamous cell carcinoma that was well differentiated and keratinizing histology. Computed tomography of her chest, abdomen, and pelvis revealed a 5 cm cervical process extending up to the upper two-thirds of her vagina and the both parameters with multiple para-aortic and pelvic lymph nodes. There was no evidence of distant metastasis. She was staged as stage IIIB according to the FIGO staging system.

**Fig. 1:**
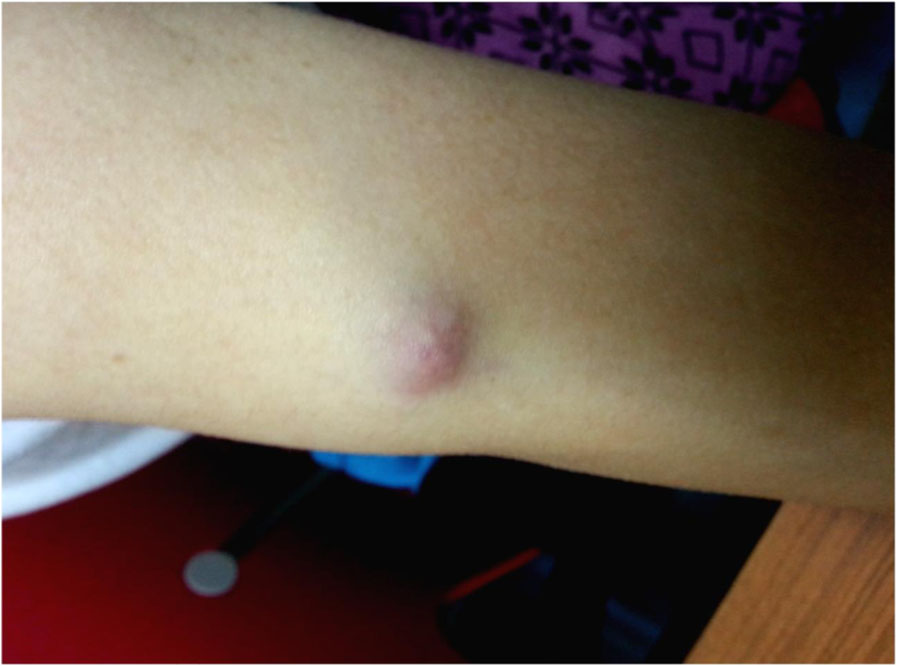
Cutaneous metastatic nodule in the upper extremity

**Fig. 2:**
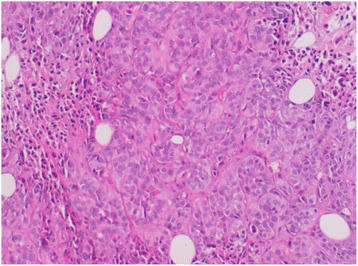
Skin biopsy at 10× magnification (hematoxylin and eosin stain), showing carcinomatosis proliferation with spans and cords

**Fig. 3:**
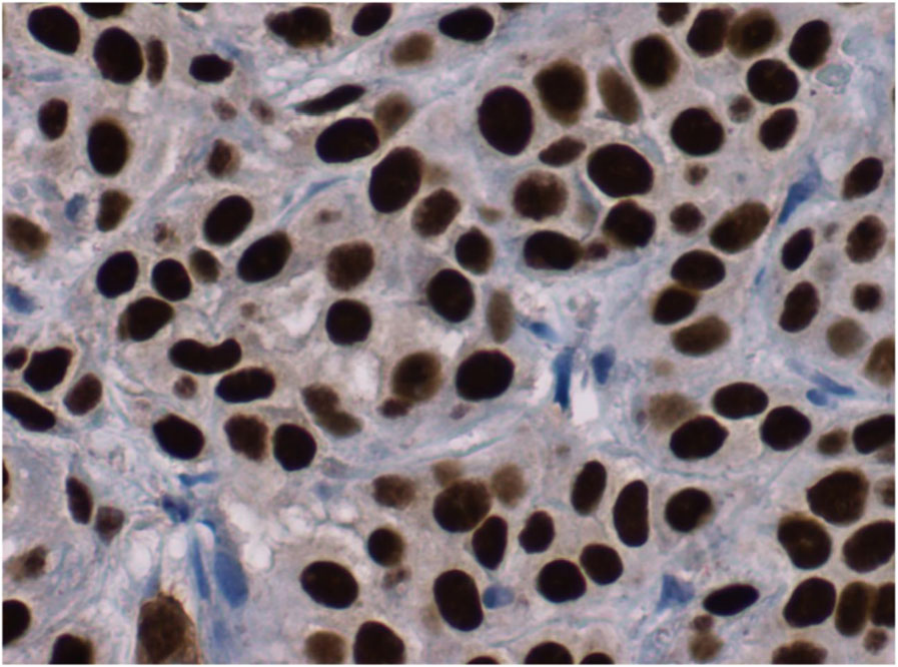
P63 immunohistochemistry performed on the skin biopsy which shows diffuse staining in the tumor cells (avidin-biotin at 40× magnification)

She was treated with concurrent chemotherapy and radiation. She received weekly cisplatin (40 mg/m^2^) and 46 Gy external beam radiation therapy to her pelvis in 23 fractions. Brachytherapy was planned before she reported the appearance of subcutaneous nodules on her arms, thighs, and chest wall (Fig. 1). A biopsy confirmed squamous cell carcinoma histology (Figs. 2 and 3).

Computed tomography of her chest, abdomen, and pelvis, and a whole body bone scan revealed multiple lung, liver, and bone metastases. She died after two courses of palliative chemotherapy, 2 months after the appearance of the skin lesions.

## Discussion

In Morocco, cervical cancer is the second most common cancer in the country and the third most common cause of death [5]. Hematogenous spread is uncommon, presenting either in a recurrence setting or initially in advanced stages; it is often localized in the liver, lung, and bone, in order of decreasing frequency [1, 2, 6].

The incidence of cutaneous metastasis from solid tumors ranges from 0.7 to 9 % [1]. The most common solid tumors metastasizing to the skin in women include breast (60 to 69 %), gastrointestinal tract (9 %), lung, and ovary [2]. Cutaneous metastases from carcinoma of the cervix are exceptional, even in the late stages of disease, with an incidence not exceeding 2 % [3, 4]. Imachi *et al*. [7] in a review of 1190 patients with cervical cancer reported that 15 patients developed skin metastasis. The incidence of skin metastasis varied according to the initial tumor stage, it was 0.8 % in stage I, 1.2 % in stage II, 1.2 % in stage III, and 4.8 % in stage IV. The incidence of skin metastasis seemed to be higher in patients with adenocarcinoma and undifferentiated carcinoma than in patients with squamous cell carcinoma.

On macroscopic examination, three common patterns of skin metastasis, such as nodules, plaques, and inflammatory telangiectasic lesions, have been reported [2, 7–9]. Lesions may be single or multiple. Our patients had multiple nodular lesions. The most common differential diagnoses include benign dermatitis, subcutaneous phycomycosis, plaque-like mycosis fungoides, and Kaposi’s sarcoma [10]. A pathological study establishes the diagnosis.

The abdominal wall and vulva are the most common sites for skin lesions, followed by the anterior chest wall [2, 11, 12]. However, cases presenting at the scalp [13, 14], extremities [15], umbilicus [16], and face [17] have been reported. In our cases, it was mainly localized in the chest and abdominal areas. The mechanism behind this type of metastases is not well established, the possible reason could be attributable to a retrograde spread of tumor secondary to lymphatic obstruction [18].

To date, no effective treatment has been identified. Treatment is nearly always palliative, and consists of using radiation, chemotherapy, or surgery, either alone or in combination [16, 19]. Electrochemotherapy (ECT) can provide immediate clinical benefit in patients with advanced cutaneous and subcutaneous metastases [20]. ECT combines the administration of a poorly permeant cytotoxic agent, such as bleomycin or cisplatin regardless of histological type, with the local application of electric pulses that induce reversible electroporation, thus improving drug diffusion into cells [21, 22]. ECT was introduced in 2006, demonstrating a high rate of efficacy and favorable toxicity profile in a European multicenter study on skin metastases from different tumor histotypes [23]. In this study, the objective response rate on treated tumor nodules was 89.0 % with complete regression in 73.3 % of cases. For our patients who had multiple metastases, we opted for palliative chemotherapy.

Skin metastasis from cervical carcinoma occurs predominantly in cases of tumor recurrences, with metastasis developing up to 10 years after initial diagnosis and averaging less than 1 year [24, 25]. In the largest series [7], the mean interval between the diagnosis of the primary tumor and skin metastasis was 16.9 months. Only two cases of cutaneous metastasis at initial presentation have been reported in the literature [12, 26]. In our report, both of the patients presented skin metastasis during the treatment or in the first months following treatment completion.

Prognosis in such cases is poor and is considered a sign for preterminal disease. The mean survival being 3 months and survival for more than 1 year seen in only 20 % of patients [7, 8, 16]. The cases that we have reported are consistent with these findings.

## Conclusions

Skin metastasis of cervical cancer remains extremely rare. Physicians should pay more attention to cutaneous lesions in patients with cervical cancer that can be the first sign of the disease, and should perform a biopsy whenever they are doubtful.

## Abbreviations

ECT: Electrochemotherapy
FIGO: International Federation of Gynecology and Obstetrics

## References

[1] Schwartz RA. Cutaneous metastatic disease. J Am Acad Dermatol. 1995;33:161–82. 10.1016/0190-9622(95)90231-7 7622642

[2] Brownstein MH, Helwig EB. Metastatic tumors of the skin. Cancer. 1972;29:1298–307. 10.1002/1097-0142(197205)29:5<1298::aid-cncr2820290526>3.0.co;2-6 4336632

[3] Brady LW, O’Neill EA, Farber SH. Unusual sites of metastases. Semin Oncol. 1977;4:59–64. 841351

[4] Rosen T. Cutaneous metastases. Med Clin North Am. 1980;64:885–900. 10.1016/s0025-7125(16)31572-3 7432046

[5] Tazi M, Benjaafar N, Er-Raki A. Incidence des Cancers a Rabat-Annee 2005. Registre des Cancers de Rabat 2009.

[6] Carlson V, Delclos L, Fletcher GH. Distant metastases in squamous-cell carcinoma of the uterine cervix. Radiology. 1967;88:961–6. 10.1148/88.5.961 6025051

[7] Imachi M, Tsukamoto N, Kinoshita S, Nakano H. Skin metastasis from carcinoma of the uterine cervix. Gynecol Oncol. 1993;48:349–54. 10.1006/gyno.1993.1061 8462901

[8] Malfetano JH. Skin metastasis from cervical carcinoma: A fatal event. Gynecol Oncol. 1986;24:177–82. 10.1016/0090-8258(86)90025-9 2940159

[9] Freeman CR, Rozenfeld M, Schopflacher P. Cutaneous metastases from carcinoma of the cervix. Arch Dermatol. 1982;118:40–1. 7059199

[10] Tharakaram S, Rajendran SS, Premalatha S, Yesudian P, Zahara A. Cutaneous metastasis from carcinoma cervix. Int J Dermatol. 1985;24:598–9. 10.1111/j.1365-4362.1985.tb05860.x 4066105

[11] Hayes AG, Berry AD. Cutaneous metastasis from squamous cell carcinoma of the cervix. J Am Acad Dermatol. 1992;26:846–50. 10.1016/0190-9622(92)70119-z 1613147

[12] Franciolini G, Momoli G, Minelli L, Mutolo F, Franchini MA, Chiodini S, et al. Cutaneous metastases from carcinoma of the cervix. Tumori. 1990;76:410–2. 10.1177/030089169007600423 2399573

[13] Shimizu I, Hayashi S, Uehara M, Nakayama S. Cutaneous metastases to the scalp from carcinoma of the uterine cervix. Arch Dermatol. 1983;119:275–6. 6838231

[14] Park JY, Lee HS, Cho KH. Cutaneous metastasis to the scalp from squamous cell carcinoma of the cervix. Clin Exp Dermatol. 2003;28:28–30. 10.1046/j.1365-2230.2003.01128.x 12558624

[15] Chen CH, Chao KC, Wang PH. Advanced cervical squamous cell carcinoma with skin metastasis. Taiwan J Obstet Gynecol. 2007;46:264–6. 10.1016/S1028-4559(08)60031-5 17962107

[16] Behtash N, Mehrdad N, Shamshirsaz A, Hashemi R, Amouzegar HF. Umbilical metastasis in cervical cancer. Arch Gynecol Obstet. 2008;278:489–91. 10.1007/s00404-008-0617-4 18379810

[17] Hsin-I Yang MD. Cellulitis-like cutaneous metastasis of uterine cervical carcinoma. J Am Acad Dermatol. 2007;56:S26–8. 10.1016/j.jaad.2006.03.028 17097380

[18] Srivastava K, Singh S, Srivastava M, Srivastava AN. Incisional skin metastasis of a squamous cell cervical carcinoma 3.5 years after radical treatment. Int J Gynecol Cancer. 2005;15(6):1183–6. 10.1111/j.1525-1438.2005.00173.x 16343209

[19] Diehl LF, Hurwitz MA, Johnson SA, Butler WM, Taylor HG. Skin metastases confined to a field of previous irradiation; report of two cases and review of the literature. Cancer. 1984;53:1864–8. 10.1002/1097-0142(19840501)53:9<1864::aid-cncr2820530912>3.0.co;2-2 6704918

[20] Campana LG, Clover AJP, Valpione S, et al. Recommendations for improving the quality of reporting clinical electrochemotherapy studies based on qualitative systematic review. Radiol Oncol. 2016;50(1):1–13. 10.1515/raon-2016-0006 PMC4825335 27069444

[21] Mir LM, Orlowski S. Mechanisms of electrochemotherapy. Adv Drug Deliv Rev. 1999;35:107–18. 10.1016/s0169-409x(98)00066-0 10837692

[22] Cabula C, Campana LG, Grilz G, et al. Electrochemotherapy in the Treatment of Cutaneous Metastases from Breast Cancer: A Multicenter Cohort Analysis. Ann Surg Oncol. 2015;22 Suppl 3:S442–50. 10.1245/s10434-015-4779-6 PMC4686551 26242370

[23] Marty M, Sersa G, Garbay JR, Gehl J, Collins CG, Snoj M, et al. Electrochemotherapy – An easy, highly effective and safe treatment of cutaneous and subcutaneous metastases: Results of ESOPE (European Standard Operating Procedures of Electrochemotherapy) study. Eur J Cancer – Suppl. 2006;4:3–13.

[24] Daw E, Riley S. Umbilical metastasis from squamous carcinoma of the cervix. Case report. Br J Obstet Gynecol. 1982;89:1666. 10.1111/j.1471-0528.1982.tb04669.x 7171520

[25] Copas RR, Spann CO, Thoms WW, Horwitz IR. Squamous cell carcinoma of the cervix metastatic to a drain. Gynecol Oncol. 1995;56(1):102–4. 10.1006/gyno.1995.1018 7821838

[26] Pertzborn S. Hematogenous skin metastases from cervical cancer at primary presentation. Gynecol Oncol. 2000;76:416–7. 10.1006/gyno.1999.5704 10684721

